# Improved CPS and CBS Extrapolation of PNO-CCSD(T) Energies: The MOBH35 and ISOL24 Data Sets

**DOI:** 10.1021/acs.jctc.3c00974

**Published:** 2024-03-21

**Authors:** Kesha Sorathia, Damyan Frantzov, David P. Tew

**Affiliations:** University of Oxford, South Parks Road, Oxford OX1 3QZ, U.K.

## Abstract

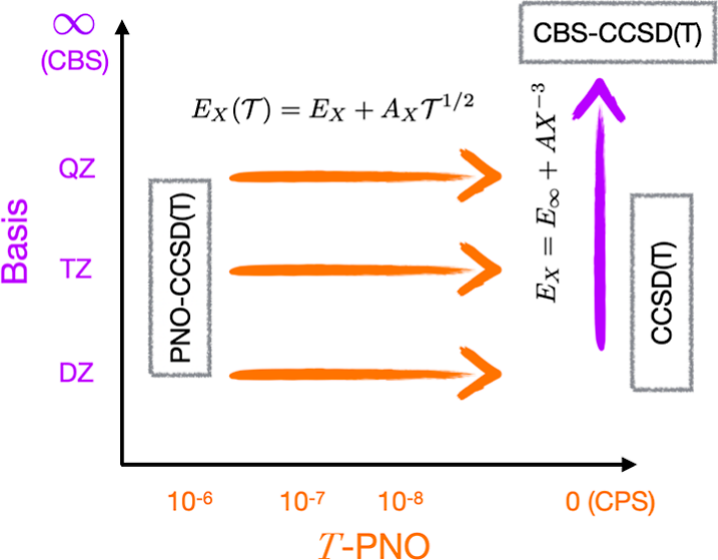

Computation of heats of reaction of large molecules is now feasible using the domain-based pair natural orbital (PNO)-coupled-cluster singles, doubles, and perturbative triples [CCSD(T)] theory. However, to obtain agreement within 1 kcal/mol of experiment, it is necessary to eliminate basis set incompleteness error, which comprises both the AO basis set error and the PNO truncation error. Our investigation into the convergence to the canonical limit of PNO-CCSD(T) energies with the PNO truncation threshold  shows that errors follow the model . Therefore, PNO truncation errors can be eliminated using a simple two-point CPS extrapolation to the canonical limit so that subsequent CBS extrapolation is not limited by the residual PNO truncation error. Using the ISOL24 and MOBH35 data sets, we find that PNO truncation errors are larger for molecules with significant static correlation and that it is necessary to use very tight thresholds of  to ensure that errors do not exceed 1 kcal/mol. We present a lower-cost extrapolation scheme that uses information from small basis sets to estimate the PNO truncation errors for larger basis sets. In this way, the canonical limit of CCSD(T) calculations on sizable molecules with large basis sets can be reliably estimated in a practical way. Using this approach, we report near complete basis set (CBS)-CCSD(T) reaction energies for the full ISOL24 and MOBH35 data sets.

## 1 Introduction

Heats of reaction and activation enthalpies computed using the coupled-cluster singles, doubles, and perturbative triples method, CCSD(T),^1^ are often accurate to within 1 kcal/mol of experimentally derived values.^2^ Even though CCSD(T) is based on a single Hartree–Fock (HF) reference wave function, the correlation treatment is complete to fourth-order in perturbation theory, and orbital relaxation is accounted for self-consistently through the singles excitations. CCSD(T) energies are frequently found to be accurate for systems where HF energies are poor, for example, in some transition metal complexes, even though they exhibit large T1-diagnostics.^3−6^

A great deal of effort has been spent on reducing the high computational cost of CCSD(T) to increase the size of system that can be modeled, for example, through massively parallel implementations,^7,8^ fragmentation methods,^9−14^ and local correlation methods.^15−19^ Local approximations exploit the short-range nature of electron correlation to reduce the scaling from  for CCSD(T) to subquadratic in system size *n*, such that calculations on very large molecules are possible,^20^ albeit with some loss of accuracy arising from the neglected contributions.^21^

This article is concerned with the domain-based pair natural orbital (PNO) approach to local correlation,^22−24^ which is particularly effective and has found widespread application in both single-reference^18,25–28^ and multireference^29–32^ correlation theories. In the PNO-CCSD(T) approach, amplitudes from MP2 theory are used to form natural orbitals for each pair of localized occupied orbitals, and the full CCSD(T) correlation treatment is performed in a truncated subset of these PNOs. The size of the subset and the corresponding error incurred is controlled through a user-defined threshold , which determines the maximum occupation number of the retained PNOs.

The increased overhead of pairwise integral transformation and nonorthogonality of PNOs between pairs is outweighed by the compression of the T2 amplitude space from  to  and the associated savings in evaluating the amplitude working equations. The integral transformation cost is also reduced to  if PNOs are confined to domains of projected atomic orbitals (PAOs) and if local density fitting is employed. Domain-based PNO-CCSD(T) has been implemented in the Turbomole,^24,33−42^ Orca,^18,22,25,43−47^ and Molpro^48−55^ program packages and is increasingly being used in studies of chemical stability and reactivity.

Martin and Semidalas have recently reported numerical studies that assess the accuracy of PNO-CCSD(T) against canonical CCSD(T) in the context of metal–organic chemistry.^56^ They find that for systems where there is moderate static correlation, the PNO truncation error can be several kcal/mol when using default thresholds of  or . By tightening the PNO threshold, the canonical result is recovered, but errors under 1 kcal/mol required very tight thresholds of . Sandler et al. have also reported sizable PNO truncation errors for reaction barriers for open- and closed-shell organic reactions when using default settings.^57^

We have previously studied the interdependence of the PNO truncation error and AO basis set error on weakly correlated systems at the level of MP2 theory.^58^ The total basis set error is the sum of the intrinsic basis set error due to the chosen AO basis and the basis set error made due to the PNO truncation. The intrinsic basis set error affects both the HF and correlation energies, whereas the PNO truncation error affects only the correlation energy. For quadruple-ζ basis sets and PNO thresholds of , we found that the PNO truncation error is commensurate with the intrinsic AO basis set error in the correlation energy. In the cases where the PNO error is dominant, increasing the basis size exhibits a false convergence, and basis extrapolation fails to recover the complete basis set limit. To reliably apply basis set extrapolation to approach the complete basis set limit, it is necessary to use energies that are closely converged to the canonical values, that is, the limit of a complete PNO space (CPS).

Care must therefore be taken to control the PNO truncation error when using PNO methods to accelerate the calculation of molecular energies, particularly for systems with moderate static correlation or when using large basis sets. Although simply tightening the PNO threshold in principle guarantees that the canonical result is recovered, the costs can increase by a factor of 2 for every 10-fold reduction in . One alternative is to exploit the systematic reduction in the PNO truncation error and use a series of calculations with decreasing  to extrapolate to the CPS limit, that is, to the canonical result. In this paper, we provide a detailed analysis of CPS extrapolation and give recommendations for best practice.

Altun et al. explored numerical fits for the behavior of the PNO truncation error with threshold *T* and proposed the error model^59^
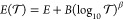
1*E* is the energy of the canonical calculation without PNO truncation and  is the energy obtained using a PNO threshold of , which is typically in the range of 10^–5^ to 10^–9^. This error model does not fit any of our data. Altun et al.,^59^ however, did not use this error model for extrapolation but instead used the general two-point extrapolation formula
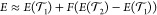
2

This approach does not specify an error model; rather, the factor *F* is determined for a chosen pair of thresholds through fitting to data. They recommend *F* = 1.5 for (6,7) and (7,8) extrapolation, independent of basis set, where (6,7) denotes extrapolation with  and .

In a simultaneous work,^58^ we proposed an error model motived by the observation that the energy is proportional to the amplitudes and that the largest discarded amplitude is proportional to the square root of the PNO truncation threshold *T*.
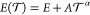
3

The exponent α is close to 0.5 but is allowed to vary with molecule and basis set because the converged amplitudes differ from the approximate semicanonical local MP2 amplitudes used to define the PNO space. We demonstrated that the resulting three-point extrapolation scheme applied to MP2 energies reduces the PNO truncation error in reaction energies equivalent to reducing the tightest PNO threshold by a factor of 50, essentially eliminating the PNO truncation error without requiring expensive calculations with very tight PNO thresholds. The three-point extrapolation formula using a sequence of thresholds  is
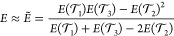
4

Our initial investigations of three-point extrapolation for PNO-CCSD and PNO-CCSD(T) energies, however, were not successful. We find that the convergence of PNO-CCSD energies with the PNO threshold does not fit the error model used for MP2 due to the differing convergence rates of the PNO-CCSD energy and the MP2-based estimate for discarded PNOs. By fixing α to the ideal value of 0.5, a two-point extrapolation formula can be applied. We find that this approach reduces the PNO truncation error by an amount equivalent to a 10-fold reduction in  for PNO-CCSD(T) energies. Using this approach, CCSD(T) near-basis-set-limit correlation energies of systems with moderate static correlation can be computed using PNO-CCSD(T) theory without incurring the high cost of very tight PNO thresholds.

Our extrapolation method is operationally very close to that of Altun et al.^59^ For a given α, two-point extrapolation using our error model results in
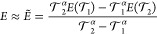
5which has the equivalent Schwenke^60^ form
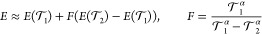
6

By choosing the factor *F* = 1.5 for (6,7) and (7,8) extrapolation, Altun et al.^59^ are in fact assuming the polynomial error model with α = 0.4771. A proper understanding of the underlying error model makes it possible to apply the extrapolation using different choices of PNO threshold, such as (6.5,7), where *F* becomes 2.366.

In this paper, we report our analysis of the PNO truncation errors in PNO-CCSD(T) theory and make recommendations for reliably extrapolating to the CPS limit to estimate the canonical CCSD(T) results. We use two data sets, the ISOL24 set of Huenerbein et al.^61^ and the MOBH35 set of Iron and Janes.^62^ The ISOL24 data set contains systems with significant dynamic correlation, up to 81 atoms, and is challenging for PNO methods because it compares energies of isomers of organic molecules with very different chemical connectivities, spatial arrangements, and long-range dispersion interactions, negating fortuitous error cancellation of local approximations. Werner and Hansen have very recently reported near-basis-set-limit isomerization energies computed using PNO-LCCSD(T)-F12b theory,^63^ which serve as a useful reference point for this work. The MOBH35 set of metal–organic barrier heights is also challenging for PNO methods since it contains systems with significant static correlation. The MOBH35 set was used by Semidalas and Martin^56^ to highlight the slow convergence of the reaction barriers with PNO threshold and larger-than-expected differences in values obtained with different implementations.

## 2 Computational Details

All calculations were performed using the TURBOMOLE program package.^20^ The structures of the ISOL24 set were taken from the Supporting Information of ref (61). We use the cc-pVDZ, cc-pVTZ, and cc-pVQZ basis sets^64^ for the PNO-CCSD(T) calculations of these molecules, which avoids the problem of internal basis set superposition errors for extended systems.

The structures for the MOBH35 test set were taken from the Supporting Information of ref (56), where the transition state structures for reactions 11 and 12 and all species of reaction 14 are modified from the original database, as recommended by Dohm et al.^65^ We use the def2-SVP, def2-TZVPP, and def2-QZVPP^66^ for PNO-CCSD(T) calculations of the MOBH35 set, which enables direct comparison to earlier estimates of CBS CCSD(T) energies for this test set. For molecules containing second- and third-row transition metal atoms, the Stuttgart relativistic effective core potentials are used.^67^

For all molecules, HF calculations were performed using the dscf program,^68^ which does not employ the density fitting approximation for the Coulomb integrals. Care was required for reactant 16 of the MOBH35 set, which converges to the incorrect state if the default extended Hückel orbital guess is applied. The PNO-CCSD(T) calculations were performed by using the pnoccsd program in TURBOMOLE V7.7. The Coulomb integrals in PNO methods are approximated using density fitting, and the corresponding Coulomb auxiliary basis sets^69,70^ are used in all cases.

The domain-based PNO-CCSD(T) implementation in TURBOMOLE uses principal domain theory,^24^ where PAO domains are selected on the basis of an approximate MP2 density in an analogous way to the PNOs themselves. The approximate MP2 density is formed in the basis of orbital specific virtuals neglecting off-diagonal Fock matrix elements in the occupied space,^38,71^ using an OSV truncation threshold linked to the PNO threshold. The CCSD amplitude equations are solved in the basis of retained PNOs, and in this work, we do not apply weak-pair approximations^72,73^ since these add additional uncertainty that complicates the analysis of the PNO truncation error. Suppression of the weak-pair approximation is achieved using the keyword multilevel off in the $pnoccsd data group. The (T) energy is computed in the basis of triple natural orbitals (TNOs)^43^ using Laplace integration,^40^ and we use a convergence threshold of 0.01 to determine the Laplace grid. All energies include a correction term that estimates the energy contribution from discarded pairs and PNOs at the level of MP2 theory, neglecting Fock coupling terms. One computational bottleneck in PNO methods is the storage of density fitting intermediates (*Q*|*ab*), which are unique to every pair *ij* and are required for the ladder terms in the CCSD equations. Despite the fact that the auxiliary functions *Q* are restricted to a pair domain in local density fitting, for large basis sets, tight PNO thresholds, and tight density fitting thresholds, the domain of functions *Q* and PNOs *a* is sufficiently large that the required disk space exceeds 1Tb. We therefore implemented the possibility to compute the integrals (*ab*|*cd*) and (*ab*|*ck*) directly without storing the three-index intermediates. This is activated by using the keyword direct. Canonical CCSD(T) calculations were computed using the ccsdf12 module of the TURBOMOLE package, using density fitting for all integrals to ensure that the canonical energies exactly correspond to the CPS limit of the PNO-CCSD(T) implementation. This is activated using the risingles and riladder keywords of the $ricc2 data group. We were able to compute canonical CCSD(T) energies for the molecules in reactions 3, 4, 6, 7, 14, 15, 16, 21, 26, 27, and 30–35 using the def2-SVP and def2-TZVPP basis sets. We denote this subset as MOBH16. We were able to compute the canonical CCSD(T)/cc-pVDZ and CCSD(T)/cc-pVTZ energies for all isomer pairs, except for 1, 4, 6, 7, 16, and 24. We denote this subset as ISOL18.

Where timings are reported, these are performed on a single Intel(R) Xeon(R) Gold 6248R CPU @ 3.00 GHz node with 48 cores, 380 Gb RAM, and 1.8Tb SSD. All computed energies are tabulated in the Supporting Information.

## 3 Extrapolation to the CPS Limit

### Error Model 

3.1

In our previous work, we showed that the error model  is very successful for PNO-MP2. The work of Altun et al.^59^ indicates that this error model with α ∼ 0.5 should also be good approximation for PNO-CCSD(T). The first questions we address in this work are (a) to what extent does this error model fit the PNO truncation error for coupled-cluster energies? and (b) to what extent does α depend on the molecule and correlation method?

**Figure 1:**
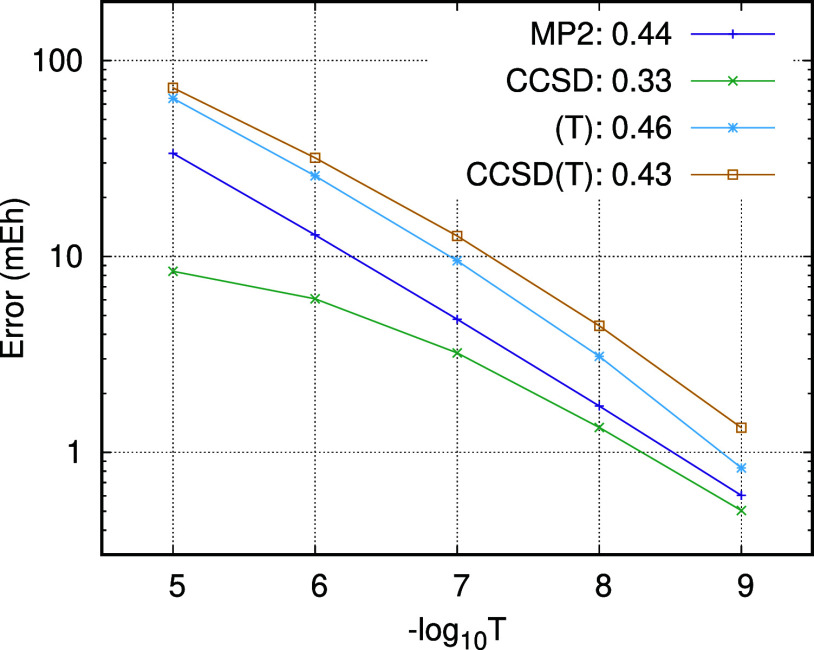
PNO truncation errors for educt number 12 of the ISOL24 set using a cc-pVTZ basis. The value of α in a best fit to  is included in the legend.

In Figure 1, we plot the PNO truncation error  against  on a log scale for the MP2, CCSD, (T), and CCSD(T) correlation energies of an example for which the canonical values are available (educt number 12 of the ISOL24 set computed using a cc-pVTZ basis). Lines of best fit using  = 10^–6^ to 10^–8^ have been computed, and the α values are given in the legend. The behavior shown for this example is typical of that seen across all the molecules in the ISOL24 and MOBH35 test sets.

In agreement with our previous findings, the PNO-MP2 truncation error follows the  error model very closely, with α = 0.44 in this case. The PNO-CCSD truncation error, on the other hand, deviates significantly from this error model, and smaller than expected errors are obtained for loose PNO thresholds. The origin of this deviation is the correction term added to the energy to account for the contribution from discarded PNOs, which is computed at the MP2 level of theory. This term overestimates the error from discarded PNOs at the CCSD level of theory and decays at a rate different to that of the CCSD energy error. Extrapolation of PNO-CCSD energies to the CPS limit by using simple one-component error models will therefore have limited success.

We turn now to the truncation error for the (T) energy. This depends on the TNO truncation threshold, which is set to be equal to the PNO threshold. We find that this contribution does follow the simple  error model. In fact, the error in the (T) energy has two sources: the TNO truncation error and the error in the T2 amplitudes used to compute the (T) energy. The error in the (T) energy is directly proportional to the TNO occupation number threshold in the same way that the error in the MP2 energy is proportional to the PNO occupation number threshold, which explains the near linearity of the log–log plot. The slight deviation from the ideal error model is a result of the error in the T2 amplitudes and follows the trend observed for CCSD. Since the TNO error is the dominant contribution to the total error in the PNO-CCSD(T) energies, extrapolation of PNO-CCSD(T) energies to the CPS limit using simple error models is expected to be successful. If in the future, the error in the (T) energy is reduced through improved TNO construction, then extrapolation of PNO-CCSD(T) to the CPS limit will become more challenging due to the increased importance of the CCSD contributions.

**Figure 2:**
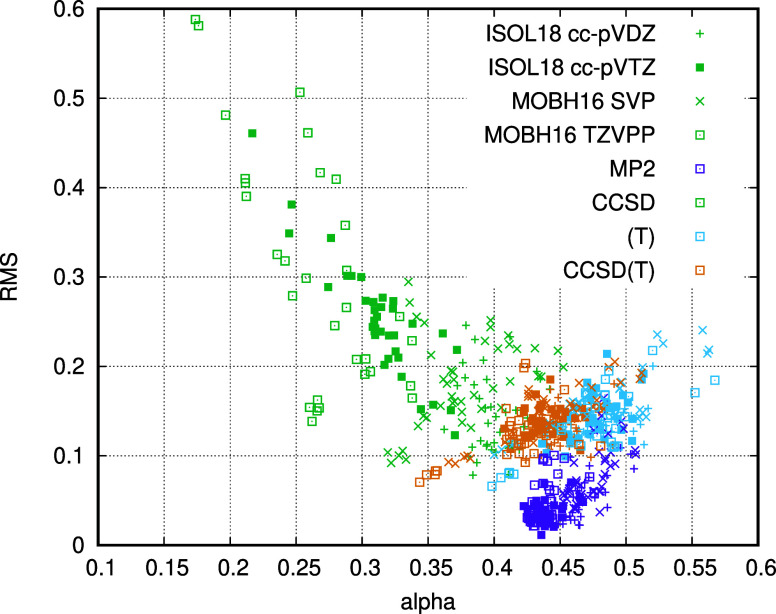
Values of α in the line of best fit to  against RMS deviation for PNO truncation errors in MP2, CCSD, and CCSD(T) energies.

For each of the molecules in our data sets where we were able to compute the canonical energies, we have performed a linear fit to the PNO truncation data using . In Figure 2, we present a scatter plot of the obtained α against the root-mean-square deviation of the data from the model. We used values of  to 10^–9^ for the fits. The data are consistent with the PNO convergence shown for educt number 12 in Figure 1. The low RMS deviations for the MP2 data indicate that the PNO-MP2 truncation follows the error model closely and the exponent α is just below the ideal value of 0.5 and is only weakly dependent on the system and basis set.

The CCSD data, however, have large RMS deviations from the model. α values ranging from 0.1 to 0.5 are obtained, reflecting varying levels of cancellation of the ring and ladder terms. The deviations are larger for the triple-ζ basis sets than the double-ζ sets, but no obvious difference is seen when contrasting the MOBH16 and ISOL18 sets. The (T) data do follow the simple error model, with modest deviations from the ideal value of α = 0.5.

The three-point extrapolation scheme we introduced in ref (58) determines the effective exponent α on a case-by-case basis from the energy convergence. For this to be accurate, the effective exponent α must be approximately constant over the range of *T* used to perform the extrapolation. Given the canonical limit *E*, the value of α corresponding to two thresholds  and  is
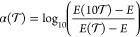
7

**Figure 3:**
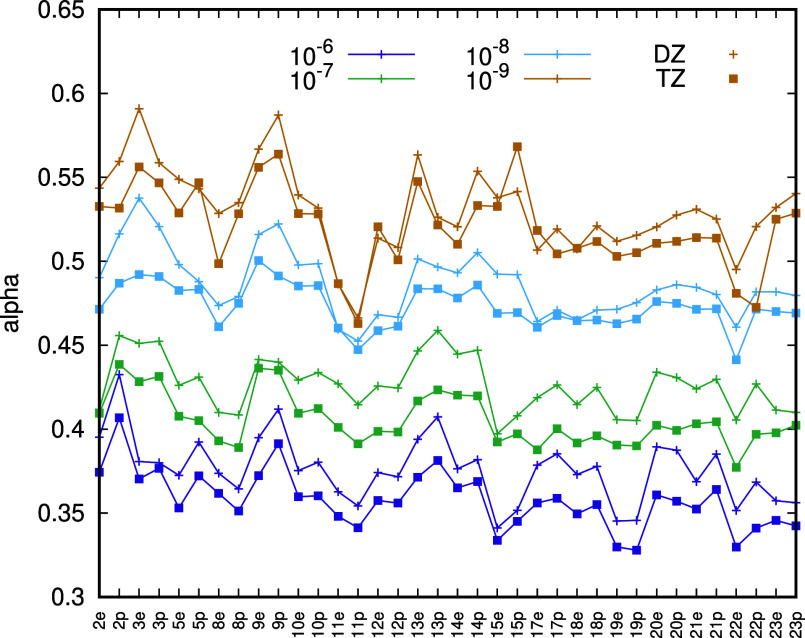
Values of  in eq 7 for PNO-CCSD(T) energies of molecules in the ISOL18 set.

In Figure 3, we display α for , 10^–7^, 10^–8^, and 10^–9^ for CCSD(T) energies of the molecules of the ISOL18 set for which we have canonical energies. Evidently, α varies considerably with , and the variation with  is larger than the variation between molecules and or between basis sets. This explains why our attempts to apply the three-point extrapolation formula to PNO-CCSD(T) energies were unsuccessful and why it is more effective to fix the exponent α close to the ideal value of 0.5 and perform a two-point extrapolation.

### Error Model 

3.2

If we fix the exponent α at the ideal value of 0.5, then the PNO truncation error can be written without loss of generality as . Two-point extrapolation assumes that the positive prefactor *A* is constant and will be accurate if  is approximately independent of . Applying the two-point extrapolation formula, we obtain
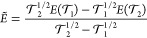
8
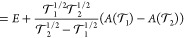
9

**Figure 4:**
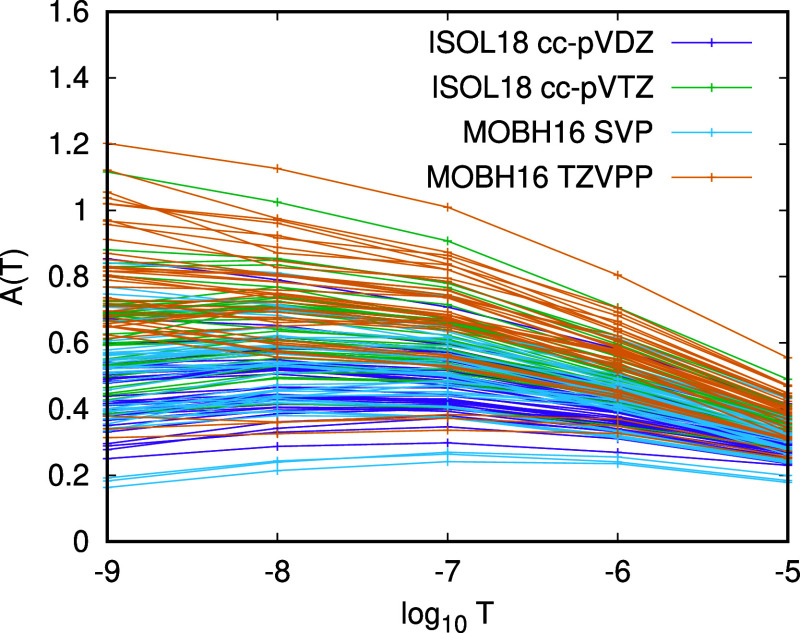
Values of  in  for PNO-CCSD(T) energies in mE_h_ per valence electron.

If *A* increases with , then the extrapolation predicts energies below the canonical limit, whereas if *A* decreases with , the correlation energy is underestimated. In Figure 4, we plot  for the molecules in our test sets where we have the canonical energies. The prefactor *A* is proportional to the number of correlated electrons in the same way as the total correlation energy, and we therefore use units of mE_h_ per valence electron for *A*. The magnitude of *A* reflects how strongly correlated the electrons are. *A* is also greater for larger AO basis sets since more of the correlation energy is recovered. Although the prefactor *A* is not constant as a function of , for most molecules, the variation is small, particularly in the range of  to 10^–8^, and we expect the two-point extrapolation to perform well.

In Table 1, we report average (AV), standard (STD), and maximum (MAX) deviations from the CPS limit for PNO-CCSD(T) energies for the ISOL18 isomerization energies and the MOBH16 barrier heights. Values with PNO threshold  to 10^–9^ are presented, together with two-point CPS extrapolation, where for example (6,7) denotes extrapolation using  and *T* = 10^–7^, respectively. The two-point CPS extrapolation of eq 5 is used with α = 0.5, which corresponds to *F* = 1.462 in eq 6.

**Table 1:** PNO-CCSD(T) Truncation Error Statistics for  to 10^–9^ in kcal/mol with and without Two-Point CPS Extrapolation

test set	basis	error	10^–6^	(5,6)	10^–7^	(6,7)	10^–8^	(7,8)	10^–9^	(8,9)
ISOL18	cc-pVDZ	AV	–0.35	–0.30	–0.19	–0.11	–0.07	–0.02	–0.02	–0.00
		STD	0.98	0.66	0.47	0.26	0.18	0.06	0.07	0.03
		MAX	2.39	–1.51	–1.14	–0.68	–0.43	–0.21	–0.18	–0.06
	cc-pVTZ	AV	–0.40	–0.33	–0.21	–0.12	–0.09	–0.04	–0.03	0.00
		STD	1.12	0.78	0.55	0.31	0.23	0.11	0.08	0.04
		MAX	–2.64	–1.86	–1.29	–0.92	–0.65	–0.42	0.22	0.09
	(DT)	AV	–0.42	–0.34	–0.22	–0.12	–0.10	–0.04	–0.03	0.00
		STD	1.19	0.83	0.58	0.34	0.26	0.14	0.09	0.05
		MAX	–2.85	–2.01	–1.36	–1.01	–0.75	–0.52	0.24	–0.11
MOBH16	def2-SVP	AV	0.28	0.16	0.10	0.02	0.03	–0.01	0.00	–0.01
		STD	0.70	0.48	0.28	0.15	0.13	0.08	0.06	0.03
		MAX	1.65	1.38	0.67	–0.54	0.33	–0.29	0.14	–0.08
	def2-TZVPP	AV	0.30	0.15	0.12	0.03	0.03	–0.00	0.01	–0.00
		STD	0.87	0.52	0.37	0.20	0.16	0.10	0.07	0.05
		MAX	–2.33	1.22	–0.89	–0.54	0.40	–0.41	0.20	–0.12
	(ST)	AV	0.31	0.14	0.13	0.04	0.04	–0.00	0.01	0.00
		STD	0.95	0.56	0.40	0.22	0.17	0.11	0.08	0.06
		MAX	–2.63	–1.21	–1.01	–0.54	0.42	–0.46	0.23	–0.14

CPS extrapolation reduces the PNO error by approximately a factor of 2, which is almost equivalent to reducing the PNO threshold by 1 order of magnitude. This observation holds for both test sets and all basis sets used. RMS errors using the default threshold of  are half a kcal/mol, with outliers around 1.5 kcal/mol. The default threshold is thus not sufficient to ensure that PNO truncation errors in energy differences are smaller than the 1 kcal/mol target of chemical accuracy. CPS (6,7) extrapolation improves this situation markedly, although the outliers are still around 1 kcal/mol. To ensure that PNO truncation errors are within chemical accuracy, it is necessary to use the very tight threshold of . With (7,8) CPS extrapolation, the maximum truncation errors for our data sets are 0.5 kcal/mol.

Table 1 also includes the corresponding values for CBS extrapolation, where we use PNO-CCSD(T) energies with two basis sets to extrapolate to the complete basis set limit. For simplicity, we use Helgaker’s two-point approach^74^ with cardinal number 2 for the def2-SVP and cc-pVDZ basis sets and 3 for the def2-TZVPP and cc-pVTZ basis sets. We observe that the PNO truncation error increases with basis size and is magnified slightly when performing CBS extrapolation due to the propagation of errors. It is therefore even more important to use tight PNO thresholds and CPS extrapolation. This underlines the conclusions of our previous work.^58^

For molecules of moderate size, we find that the cost of a PNO-CCSD(T) calculation can increase by a factor of 2–3 with every 10-fold decrease of  and increases by a factor of 2–3 with every increment in the cardinal number of the AO basis. Performing PNO-CCSD(T) calculations with large basis sets and tight thresholds is expensive and can exceed the limits of commonly available disk and memory resources. F12 explicitly correlated methods^75^ are a good solution to this computational bottleneck. It is, however, very useful to be able to access the basis set limit using regular methods.

One approach to reducing the PNO truncation error of PNO-CCSD(T) calculations with a large basis is to estimate the error using a smaller basis set or a lower cost method and add a correction term.^76,77^ This assumes that the PNO truncation error is approximately constant across methods and basis sets, but, as we have previously noted, the prefactor  in fact has a significant basis set dependence. It has an even larger variation with the correlation method since different proportions of the correlation energy are recovered.

**Figure 5:**
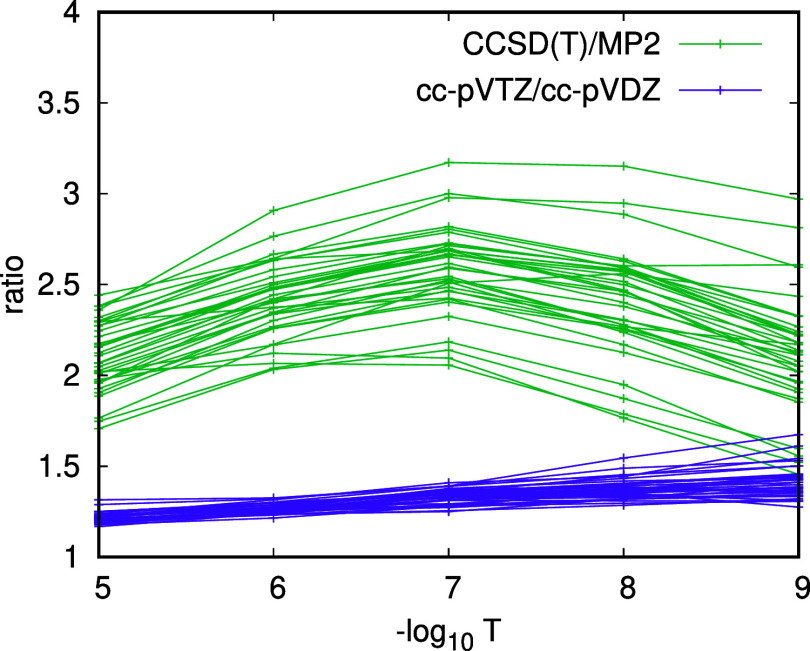
Ratios of  between the cc-pVTZ and cc-pVDZ basis sets and between the CCSD(T) and MP2 methods for the ISOL18 set.

However, we find that the ratio between the  for different basis sets is only weakly dependent on . To a lesser extent, the variation in the ratio between  for different methods is also relatively small. This is seen from Figure 5 where we plot the ratio between  for the cc-pVDZ and cc-pVTZ basis sets for the molecules of the ISOL18 set, together with the ratio between  for the CCSD(T) and MP2 correlation energies in the cc-pVTZ basis. We can therefore accurately estimate the scaling factor that relates the PNO truncation error for one method or basis set with another
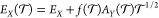
10
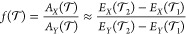
11Here, *X* denotes an expensive method and basis set combination, and *Y* denotes a less demanding approach. Since *f* is only weakly dependent on , it can be computed using relatively loose PNO thresholds with low cost. Applying two-point extrapolation leads to the following simple formula for the CPS limit for method *X*
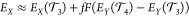
12
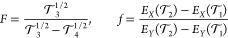
13

The PNO truncation thresholds should be chosen such that . If , then five calculations are required in total.

We have tested the accuracy of eq 12 for the CCSD(T)/cc-pVTZ isomerization energies of the ISOL18 set. In Table 2, we report deviations from the CPS limit for different choices of method *Y*. The notation (5,6,7) refers to , , , etc. For comparison, the values obtained with *Y* = CCSD(T)/cc-pVTZ are also listed, which are identical to those obtained by simply applying eq 6 with  and .

**Table 2:** PNO Truncation Error Statistics for CCSD(T)/cc-pVTZ Isomerization Energies of the ISOL18 Set Using Equation 12

*Y*	error	(5,6,7)	(6,7,8)	(7,8,9)
CCSD(T)/cc-pVTZ	AV	–0.12	–0.03	0.00
	STD	0.31	0.11	0.04
	MAX	–0.92	–0.42	0.09
CCSD(T)/cc-pVDZ	AV	–0.09	–0.00	–0.02
	STD	0.32	0.13	0.07
	MAX	–0.86	–0.35	–0.24
MP2/cc-pVTZ	AV	–0.19	–0.11	–0.01
	STD	0.83	0.23	0.14
	MAX	2.31	–0.71	–0.42
MP2/cc-pVDZ	AV	–0.14	–0.11	0.01
	STD	0.79	0.30	0.12
	MAX	1.76	–0.70	–0.43

Comparing the CCSD(T)/cc-pVTZ and CCSD(T)/cc-pVDZ results, we see that the accuracy is very similar. Therefore, to obtain (7,8) quality PNO-CCSD(T)/cc-pVTZ values, it is not necessary to perform PNO-CCSD(T)/cc-pVTZ calculations with  and 10^–8^. Instead,  and 10^–7^ are required, together with the significantly cheaper , 10^–7^, and 10^–8^ calculations using the smaller cc-pVDZ basis.

Comparing the CCSD(T)/cc-pVTZ and MP2/cc-pVTZ results, we see that there is a marked reduction in accuracy when using MP2. The variation of the factor *f* in eq 12 with *T* is greater and less systematic when changing method than changing basis, and the uncertainty in the extrapolated energies is correspondingly larger. Since PNO-MP2 calculations are much less expensive than PNO-CCSD(T) calculations, it is nevertheless potentially worthwhile to use (6,7,8) with MP2 since the results are a slight improvement over (6,7) without the MP2 correction. Reducing both the method and the basis set introduces errors that are too large and is not recommended.

In Table 3, we present PNO truncation errors for reaction 13 of the MOBH35 set as an example of a system with large static correlation and slow PNO convergence. We compare different schemes for reducing the PNO truncation error of PNO-CCSD(T)/def2-TZVPP energies: no extrapolation; adding an MP2 correction Δ as advocated by Kubas;^77^ scaled extrapolation using the def2-SVP basis; and straightforward two-point extrapolation. For each method, we report the sum of the wall times taken to estimate the canonical energy of the reactant. All timings include the HF calculation, which took 12 min using density fitting. Although adding a correction Δ = *E*_MP2_ – *E*_PNO-MP2_ does reduce the PNO truncation error for loose thresholds, with minimal additional expense, it is rather ineffective for tight thresholds. The most cost-effective way to ensure that the canonical result is recovered is to use the (6,7,8) scaled extrapolation scheme, which avoids the expense of performing a PNO-CCSD(T)/def2-TZVPP calculation with a very tight threshold of 10^–8^.

**Table 3:** Timings and PNO Truncation Errors in kcal/mol of CCSD(T)/def2-TZVPP Barrier Heights for MOBH35 Reaction 13 Using PNO Thresholds 10^–6^ to 10^–8^ and with Extrapolation and Correction Schemes

scheme	forward	reverse	min
6	5.5	3.1	92
6 + Δ	3.7	2.2	93
(5,6)	3.7	1.9	144
7	2.5	1.4	184
7 + Δ	1.8	1.1	185
(5,6,7)	1.0	1.0	213
(6,7)	1.1	0.6	265
8	0.9	0.5	554
8 + Δ	0.6	0.4	555
(6,7,8)	0.1	0.3	398
(7,8)	0.1	0.1	726

## 4 Benchmark Data

### 4.1 MOBH35

Our CPS extrapolation approach makes it possible to reliably estimate the canonical CCSD(T) energies of large systems with large basis sets using PNO methods and thus extrapolate to the CBS limit without being limited by PNO truncation errors. In Table 4, we report our best estimates for the canonical CCSD(T) barrier heights of the full MOBH35 set. Our def2-SVP values agree closely with those previously reported, and we present, for the first time, def2-QZVPP values for the full set, including reactions 17–20, which were omitted from the work of Semidalas and Martin.

**Table 4:** Best Estimates for CCSD(T) Barrier Heights for the MOBH35 Test Set in kcal/mol

	forward					reverse				
rxn	SVP	TZVPP	(ST)	QZVPP	(TQ)	SVP	TZVPP	(ST)	QZVPP	(TQ)
1	27.06	26.67	26.43	26.26	25.94	14.02	13.91	13.87	14.45	14.75
2	5.62	5.91	5.62	5.84	5.77	25.10	22.25	21.87	22.34	22.43
3	0.95	1.03	0.90	1.01	1.01	27.07	26.14	26.80	27.09	27.69
4	2.35	1.54	0.98	1.45	1.38	8.60	7.87	8.34	8.43	8.82
5	4.43	4.69	4.28	4.94	5.03	22.09	22.73	22.60	22.64	22.68
6	13.48	15.60	15.81	15.84	15.98	13.46	14.82	14.32	15.02	14.98
7	26.62	27.70	28.01	27.72	27.80	18.26	18.91	18.29	18.91	18.85
8^a^	37.28	35.65	35.69	36.01	35.70	32.77	32.54	32.47	33.46	33.70
9^a^	28.97	28.08	28.50	29.42	30.01	14.90	12.07	10.66	11.09	10.82
10	–3.52	–3.82	–3.79	–4.16	–4.43	9.59	8.95	7.41	8.03	7.56
11^a^	29.89	30.05	29.91	29.59	29.39	84.13	83.17	83.10	82.95	82.54
12	5.68	5.37	5.46	5.31	5.28	36.95	37.38	38.33	37.17	37.19
13	18.85	20.69	21.37	20.88	21.24	48.37	48.59	48.89	48.52	48.42
14	10.20	10.26	10.26	10.31	10.35	13.33	14.43	13.95	14.89	14.99
15	23.84	20.77	19.77	20.44	20.14	74.62	74.91	74.55	75.96	76.51
16	37.24	35.09	34.50	35.04	34.89	55.45	53.61	53.84	53.93	54.37
17^a^	24.22	22.84	24.20	21.28	20.54	29.94	28.35	27.54	30.94	31.87
18^a^	25.53	26.12	27.79	24.94	24.44	27.93	28.35	27.83	31.70	33.09
19^a^	11.05	11.55	13.09	10.96	10.58	30.36	28.80	27.98	31.69	32.85
20^a^	7.60	10.49	12.28	10.29	10.06	28.15	28.71	28.20	32.18	33.63
21	11.06	8.14	7.87	8.44	8.70	11.06	8.15	7.88	8.44	8.69
22	14.90	14.42	13.64	14.39	14.37	30.83	27.25	26.42	27.66	28.00
23	29.40	29.98	28.81	29.90	29.85	20.82	20.45	20.12	20.64	20.79
24^a^	1.08	2.44	2.35	2.71	2.85	18.61	17.03	17.34	16.61	16.54
25^a^	1.52	2.78	2.60	3.11	3.23	14.58	12.90	13.08	12.91	13.08
26	21.79	25.07	25.94	25.25	25.39	–0.07	0.13	0.22	0.14	0.17
27	16.11	14.09	14.00	13.87	13.84	1.29	1.88	1.75	2.20	2.42
28	32.00	30.63	30.50	30.21	29.86	16.85	15.94	15.41	15.84	15.81
29	15.76	15.30	15.05	14.91	14.69	33.87	32.02	30.74	31.35	30.88
30	10.94	10.07	9.94	9.81	9.64	19.89	17.10	16.29	17.19	17.15
31	2.37	3.41	4.00	3.26	3.07	12.31	13.35	13.03	13.01	12.79
32	23.56	20.43	20.31	20.01	19.87	58.31	62.06	62.67	63.31	64.08
33	2.76	1.21	0.70	1.13	0.99	10.00	8.24	8.21	8.10	8.02
34	28.85	29.82	29.56	29.11	28.66	4.38	3.54	3.49	3.24	2.99
35	15.00	16.68	16.51	17.54	17.99	–3.74	–2.42	–2.24	–2.08	–1.86

a def2-QZVPP values are computed using the (6,7,8) threshold combination with *Y* = def2-TZVPP, rather than using the (7,8,9) threshold combination with *Y* = def2-SVP.

To compute the CPS limit for CCSD(T)/def2-SVP, we use (8,9) extrapolation of PNO-CCSD(T) energies based on the  convergence model, which has an RMS deviation from the canonical limit of under 0.1 kcal/mol. PNO-CCSD(T)/def2-SVP calculations using  were possible for the full data set using the Turbomole implementation. Disk space limitations precluded the use of  with the def2-TZVPP, but  was possible for all molecules. To estimate the CCSD(T)/def2-TZVPP canonical limit, we use (7,8,9) extrapolation
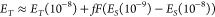
14
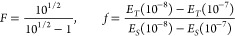
15

It was also possible to compute PNO-CCSD(T)/def2-QZVPP values for all but the largest molecules, and we also used the (7,8,9) extrapolation with *Y* = def2-SVP. For the largest molecules, we used (6,7,8) extrapolation with *Y* = def2-TZVPP
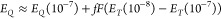
16
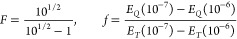
17

Our def2-SVP values do not agree perfectly with the subset of canonical values reported by Semidalas and Martin. Their data are based on HF energies computed using density fitting, whereas we did not employ this approximation in our HF calculations, and the difference in the HF energies and the resulting change in the correlation energies amounts to 0.1 kcal/mol deviations on average. Reactions 5, 6, 12, 24, 25, 26, 31, 32, and 35 have deviations between 0.1 and 0.3 kcal/mol, which is not untypical.^78^ In addition, Table 4 reports (8,9) extrapolated CPS values rather than canonical values. Although these are within 0.1 kcal/mol of the canonical barrier heights for the MOBH16 set, this does not contain reactions 8, 9, and 13, which are more strongly correlated and converge more slowly with the PNO threshold. Residual CPS errors of 0.4 kcal/mol may remain for these reactions. A conservative error bar of 0.2 kcal/mol should be placed on the PNO estimates for the canonical values, except for reactions 8, 9, and 13, which may have errors of 0.5 kcal/mol.

The primary difference in our best CBS estimate from that of Semidalas and Martin is that we have performed a (TQ) extrapolation for both the CCSD and (T) correlation energies. Semidalas and Martin did not compute the (T) contribution using the def2-QZVPP basis and instead used a (ST) extrapolation for the (T) energy. Nevertheless, our barrier heights differ by less than 0.5 kcal/mol from their values for all reactions except for 8, 9, and 13. For these more strongly correlated systems, our values differ by up to 2.5 kcal/mol and in fact lie between those of Semidalas and Martin^56^ and the original values reported by Iron and Janes.^62^

### 4.2 ISOL24

Near-CBS data for the ISOL24 set have very recently been computed by Werner and Hansen using the PNO-LCCSD(T)-F12b method in Molpro and a modified aug-cc-pVQZ basis set. In Table 5, we compare their isomerization energies with our values computed using CBS extrapolation of CPS-extrapolated PNO-CCSD(T) energies.

**Table 5:** CPS- and CBS-Extrapolated CCSD(T) Isomerization Energies for the ISOL24 Test Set in kcal/mol

iso	DZ	TZ	(DT)	QZ	(TQ)	F12^a^
1	67.35	70.22	70.71	71.24	71.55	71.53
2	40.78	39.14	39.90	38.59	38.68	38.13
3	4.29	8.76	9.70	9.67	10.21	10.29
4	75.61	71.55	73.90	70.17	69.89	69.03
5	34.76	32.51	32.92	32.65	33.02	32.85
6	18.62	22.41	23.44	23.47	24.07	24.14
7	18.90	17.92	18.46	17.83	17.90	17.72
8	23.04	24.00	23.13	24.24	24.01	23.93
9	22.77	21.68	21.63	21.36	21.13	21.10
10	6.07	6.14	6.37	6.43	6.47	6.26
11	35.66	35.45	35.19	36.18	36.65	36.80
12	0.68	0.43	0.43	0.42	0.48	0.42
13	29.00	32.25	32.84	33.03	33.29	33.20
14	4.26	4.68	5.30	4.97	5.06	4.92
15	10.82	4.37	3.56	4.03	4.15	4.07
16	20.68	22.69	23.61	22.76	23.04	22.62
17	9.51	9.19	9.57	9.29	9.45	9.59
18	27.06	24.78	24.60	24.19	23.93	23.77
19	18.77	18.41	18.06	18.41	18.37	18.28
20	6.18	5.37	4.98	5.18	4.95	5.13
21	11.17	11.00	11.51	11.29	11.60	11.38
22^b^	–3.59	–0.98	–1.25	0.09	0.18	1.08
23	24.04	23.93	24.31	23.72	23.75	23.66
24	16.32	15.58	15.49	15.34	15.25	15.25

a PNO-LCCSD(T)-F12b/APVQZ’ values taken from ref (63).

b Product and educt reversed to maintain positive sign.

We were able to compute PNO-CCSD(T) energies with  for the cc-pVDZ basis and  for the cc-pVTZ and cc-pVQZ basis sets for all molecules. Our best estimate of canonical CCSD(T) energies is therefore obtained using an (8,9) extrapolation for the cc-pVDZ basis and a (7,8,9) extrapolation with *Y* = cc-pVDZ for the cc-pVTZ and cc-pVQZ basis sets.

Our best CBS values are from (TQ) extrapolation. The (TQ) and F12 isomerization energies agree to within 0.5 kcal/mol for all isomer pairs, except for 4 and 22, where the difference is 0.9 kcal/mol. This level of agreement is only slightly worse than that expected from canonical theory, and these results underline the viability of using PNO-CCSD(T) theory in CBS extrapolation, provided that the PNO truncation error can be properly controlled. The larger deviations of the CBS estimates for 4 and 22 compared to F12 arise not from the PNO errors but from the lack of diffuse functions in the basis set used.

In Table 6, we report the results of equivalent calculations using aug-cc-pVXZ′ (APVXZ′) basis sets, where the prime denotes that diffuse functions are removed from the H atoms. For these calculations, the multilevel approximation was turned on. The combined CPS and CBS extrapolation approach is equally applicable when using diffuse functions and when using the multilevel approximation, and the results using diffuse functions for these cases are much closer to the F12 results.

**Table 6:** CPS- and CBS-Extrapolated CCSD(T) Isomerization Energies in kcal/mol Using APVXZ′ Basis Sets

iso	DZ	TZ	(DT)	QZ	(TQ)	F12^a^
4	85.55	74.45	74.19	70.94	68.63	69.03
22^b^	–5.94	–1.96	–1.88	0.00	0.97	1.08

a PNO-LCCSD(T)-F12b/APVQZ′ values taken from ref (63).

b Product and educt reversed to maintain positive sign.

## 5 Conclusions

Domain-based PNO-CCSD(T) theory provides a low-scaling approximation to canonical CCSD(T) theory that makes it possible to perform accurate calculations on large molecular systems with  atoms. However, for such calculations to achieve the so-called “gold standard” status and be used to predict reaction enthalpies to within 1 kcal/mol of experiment, it is necessary to ensure that both the AO basis set truncation error and the PNO truncation error are sufficiently converged.

The smooth convergence of the correlation energies with basis set size for canonical theories is well documented to follow an *E*_*X*_ = *E*_∞_ + *CX*^–3^ basis set error model with the cardinal number *X*, and extrapolation to the CBS limit is routinely applied. In this article, we have demonstrated that the PNO truncation error for the CCSD(T) energy follows  with PNO truncation threshold , so that the combined convergence is . The prefactor *A*_*X*_ is basis set dependent, being greater for larger basis sets, proportional to the number of correlated electrons, and larger for more strongly correlated systems.

To accurately obtain CBS CCSD(T) energies using PNO methods, the most reliable approach is to first eliminate the PNO truncation error through CPS extrapolation to obtain the canonical energies *E*_*X*_ and *E*_(*X*+1)_ and to perform two-point extrapolation in the usual manner. CPS extrapolation to the canonical limit proceeds in a way analogous to CBS extrapolation and requires calculations with two PNO thresholds; typically, we chose 10*T* and *T*. We find that for systems with moderate static correlation, extrapolation using “tight” PNO thresholds of  is not sufficient to ensure that the PNO truncation errors are less than 1 kcal/mol. Reliable results are, however, obtained for all cases in the ISOL24 and MOBH35 data sets when extrapolating using  and .

Regarding the basis set, it is well documented^2^ that basis set errors in CCSD(T) calculations using double-ζ quality basis sets are commensurate with the uncertainties in density functional approximations and that (TQ) extrapolation is the minimum required to achieve “gold standard” results. The combination of tight PNO thresholds and large basis sets places a heavy burden on current PNO-CCSD(T) implementations, particularly in the I/O of pair-specific integrals stored on disk. We have found that the prefactor *A*_*X*_ for a large basis set can be accurately estimated using information from PNO calculations using a smaller basis set and that the most expensive calculations in the extrapolation procedure can be avoided with very little loss of accuracy.

Our recommended CPS extrapolation approach to obtain *E*_*X*_, the CCSD(T) energy in basis set with cardinal number *X*, is to use
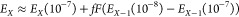
18
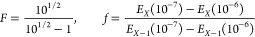
19

## Supporting Information Available

All energies computed in this work (XLSX)
